# Artificial intelligence optimizes the standardized diagnosis and treatment of chronic sinusitis

**DOI:** 10.3389/fphys.2025.1522090

**Published:** 2025-03-13

**Authors:** Yang-Yang Liu, Shao-Peng Jiang, Ying-Bin Wang

**Affiliations:** Department of Otolaryngology, First Affiliated Hospital of Gannan Medical University, Ganzhou, China

**Keywords:** artificial intelligence, machine learning, deep learning, chronic sinusitis, diagnosis, treatment

## Abstract

**Background:**

Standardised management of chronic sinusitis (CRS) is a challenging but vital area of research. Not only is accurate diagnosis and individualised treatment plans required, but post-treatment chronic disease management is also indispensable. With the development of artificial intelligence (AI), more “AI + medical” application models are emerging. Many AI-assisted systems have been applied to the diagnosis and treatment of CRS, providing valuable solutions for clinical practice.

**Objective:**

This study summarises the research progress of various AI-assisted systems applied to the clinical diagnosis and treatment of CRS, focusing on their role in imaging and pathological diagnosis and prognostic prediction and treatment.

**Methods:**

We used PubMed, Web of Science, and other Internet search engines with “artificial intelligence”、“machine learning” and “chronic sinusitis” as the keywords to conduct a literature search for studies from the last 7 years. We included literature eligible for AI application to CRS diagnosis and treatment in our study, excluded literature outside this scope, and categorized it according to its clinical application to CRS diagnosis, treatment, and prognosis prediction. We provide an overview and summary of current advances in AI to optimize the diagnosis and treatment of CRS, as well as difficulties and challenges in promoting standardization of clinical diagnosis and treatment in this area.

**Results:**

Through applications in CRS imaging and pathology diagnosis, personalised medicine and prognosis prediction, AI can significantly reduce turnaround times, lower diagnostic costs and accurately predict disease outcomes. However, a number of challenges remain. These include a lack of AI product standards, standardised data, difficulties in collaboration between different healthcare providers, and the non-interpretability of AI systems. There may also be data privacy issues involved. Therefore, more research and improvements are needed to realise the full potential of AI in the diagnosis and treatment of CRS.

**Conclusion:**

Our findings inform the clinical diagnosis and treatment of CRS and the development of AI-assisted clinical diagnosis and treatment systems. We provide recommendations for AI to drive standardisation of CRS diagnosis and treatment.

## 1 Introduction

Rhinosinusitis is an inflammatory disease that affects the nasal mucosa and sinus mucosa mainly caused by viral and bacterial infections, and is mainly manifested by symptoms such as nasal congestion, runny nose, postnasal drip, loss of smell, head and face pain or pressure (Bleier and Paz-Lansberg, 2021). Acute rhinosinusitis (ARS) lasts less than 12 weeks and becomes chronic rhinosinusitis (CRS) when it lasts longer than 12 weeks (Fokkens et al., 2012). CRS is a common disease worldwide, with an incidence rate of about 8% in China, 2.1%–13.8% in the United States, and 6.9%–27.1% in Europe (Liu et al., 2020; Orlandi et al., 2021). Nasal symptoms, reduced sleep quality and fatigue caused by CRS seriously affect the quality of life of patients and impose a heavy burden on society and the economy (Van Crombruggen et al., 2011). CRS develops in all age groups and has a complex etiology that is the result of a combination of factors. Patients with CRS can get relief from their symptoms with medication, but most patients with CRS cannot be cured with medication, and surgery is currently an important treatment for chronic sinusitis (Ghogomu and Kern, 2017). The key to the surgical treatment of chronic sinusitis is to repair the normal structure and function of the nasal mucosa, and the implementation of surgery can not only play a therapeutic role, but also restore the morphology of the sinus mucosa and reconstruct sinus ventilation and drainage through limited or small-scale surgery, so that most patients with sinusitis can be treated, and the clinical application value is high (Chandra et al., 2016). But surgical treatment still has a high recurrence rate (DeConde et al., 2017),and the treatment protocols for surgical treatment of chronic sinusitis have not yet been standardised.

With the development and innovation of artificial intelligence (AI) in recent years, the “AI + medical” model has gradually emerged, and AI has been well applied in various medical aspects such as disease prediction (Shu et al., 2021), disease diagnosis and treatment (Huang et al., 2023), and new drug development (Vatansever et al., 2021). Similarly, AI is widely used in otolaryngology head and neck surgery (Alter et al., 2024). This review focuses on the current state of research on AI in chronic rhinosinusitis by presenting its prospects for clinical applications in the diagnosis, treatment and prognostic assessment of chronic rhinosinusitis and discussing how to optimise the standardised diagnosis and treatment of chronic rhinosinusitis through AI.

## 2 Artificial intelligence technology

**FIGURE 1:**
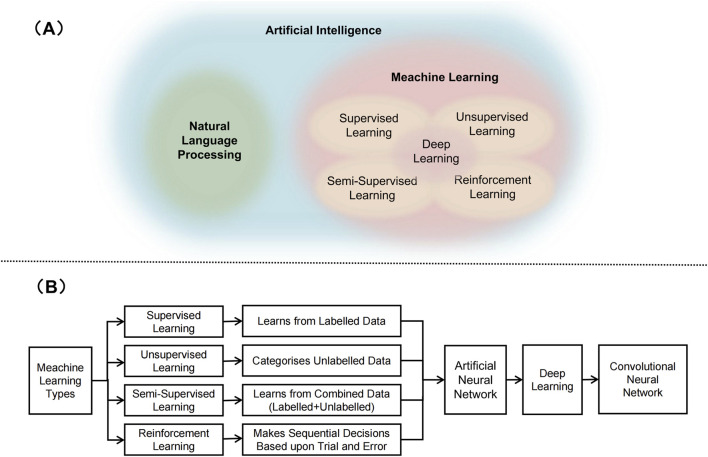
Artificial intelligence related technologies. **(A)** Schematic representation of relation between artificial intelligence, natural language processing, meachine learning, and deep learning. **(B)** Various types of machine learning techniques.

AI is a modern approach based on computer science that develops programmes and algorithms to enable devices to intelligently and efficiently perform tasks that would normally require skilled human operation (Manickam et al., 2022) (Figure 1). Machine learning (ML) is the most widely used AI method. According to the algorithm structure and learning method, ML can be further classified into supervised learning (SL), semi-supervised learning (sSL), unsupervised learning (uSL) and intensive learning (IL) (Mohammed et al., 2016) (Figure 1B).In SL, algorithms are trained using input data and machine learning algorithms learn from the training and can be used to predict possible future events (Han et al., 2022). USL methods can identify patterns in each dataset, even if the data is not correctly classified or labelled (Han et al., 2022). This is widely used for extracting generative features, identifying meaningful trends and structures, grouping results, and exploratory purposes. SSL is in between ‘unsupervised’ and ‘supervised’ learning, as it works on both labelled and unlabelled data (Han et al., 2022). Therefore, its ultimate goal is to provide better predictions than those produced using only labelled data in the model. IL is a powerful tool used to train AI models that can help improve the operational efficiency of automating or optimising complex systems (Kaelbling et al., 1996). Deep learning (DL) is part of a broader family of representation learning ML methods based on artificial neural networks (ANN) (Han et al., 2022). DL provides a computational architecture by combining multiple processing layers (e.g., input, hidden, and output layers) to learn from data. The main advantage of deep learning over traditional machine learning methods is that it offers better performance in a number of situations, especially learning from large datasets (Sarker et al., 2020). Convolutional neural networks (CNN) augment standard ANNs designed to process data with a grid-like structure (e.g., images) (Valueva et al., 2020). Although ANNs have a greater computational burden, they do not require any human intervention, and they have the advantage of automatically detecting important features, so CNNs are considered more powerful than traditional ANNs.

## 3 Artificial intelligence in chronic sinusitis

### 3.1 Artificial intelligence-assisted diagnosis of chronic sinusitis

#### 3.1.1 Application of artificial intelligence in imaging diagnosis of chronic sinusitis

Imaging is one of the main means of clinical diagnosis of CRS, and the Lund-MacKay score and the global osteitis scoring scale (GOSS), which is commonly used in clinical practice to assess the bony changes in the sinuses, both require CT examination (Georgalas et al., 2010). However, there was a clear inconsistency between the imaging reports and the clinical care concerns, with significant differences between reports from different radiologists. Moreover, current scoring systems can only achieve semi-quantitative assessment, and detailed preoperative assessment is time-consuming. Therefore, an automatic quantitative assessment system that is objective and rapid needs to be developed. With the development of AI, most scholars based on AI to assist the diagnosis through the big data of imaging data (e.g., sinus X-ray film, sinus CT) (Table 1).

**TABLE 1:** Application of artificial intelligence in the imaging diagnosis of chronic sinusitis.

Researcher	Year	System	Function	AI	Ref
Kim	2019	ResNet	Diagnosis of the maxillary sinusitis on Waters’ view radiographs	DL	Kim et al. (2019a)
Kim	2019	VGG-16 VGG-19 ResNet-101	Identification of the maxillary sinus on paranasal sinus X-ray images	DL	Kim et al. (2019b)
Jeon	2021	Detector (Mdet) Classifier (Mcls)	Diagnosis of frontal sinusitis, ethmoid sinusitis, and maxillary sinusitis on Waters and Cardwell views	DL	Jeon et al. (2021)
Chowdhury	2019	Inception-V3	Automated classification of OMC inflammation	CNN	Chowdhury et al. (2019)
Massey	2022	CNN Model	Automated CT analysis for quantitative sinus opacity	DL	Massey et al. (2022)
Qi	2021	Leaky ReLU	Improves the performance of maxillary sinus segmentation	CNN	Qi et al. (2021)
Choi	2022	U-Net	Fully automated segmentation of the maxillary sinuses for more accurate results	DL	Choi et al. (2022)
Morgan	2022	3D U-Net	Automatically segment and create a 3D virtual model of the maxillary sinus from CBCT images	CNN	Morgan et al. (2022)
Whangbo	2024	Normal/Dense/Residual/Residual-Dense 3D U-Net	Multi-class segmentation of sinuses in patients with sinusitis	CNN	Whangbo et al. (2024)

DL, deep learning; CNN, convolutional neural network; OMC, osteomeatal complex; CBCT, cone-beam computed tomographic.

Sinuses often overlap with other craniofacial bones on radiographs, leading to a high rate of false-negative diagnoses (Hagiwara et al., 2022). To address this issue, in 2019 Kim et al. (Kim Y. et al., 2019) trained a ResNet model to diagnose maxillary sinusitis on Vaishnavian position X-rays, and the area under the curve (AUC) on the time-validated and place-validated sets was 0.93 and 0.88, respectively, which was significantly higher than that of radiologists. In the same year Kim et al. (Kim HG. et al., 2019) trained various models such as VGG-16,VGG-19 and ResNet-101 to classify the Vaishnavite bit radiographs respectively and finally diagnosed them using majority decision algorithm, which showed a validation set accuracy of 94.12% with an AUC of 0.942. In 2021, Jeon et al. (Jeon et al., 2021) used a multi-view CNN, fusing information from Vaishnavian and Koch’s position X-rays for classification, and their AUCs for the diagnosis of sieve sinusitis and maxillary sinusitis were 0.78 and 0.88, respectively, which were higher than that of radiologists.

In 2019 Chowdhury et al. (Chowdhury et al., 2019) explored the feasibility of using a CNN to automatically identify clinically relevant information from sinus CT scans, and classified sinonasal complexes from coronal CT images of the paranasal sinuses of 239 patients with chronic rhinosinusitis, achieving an 85% accuracy rate in classifying sinonasal complexes as either ‘open’ An accuracy of 85 per cent was achieved in classifying the sinonasal complex as ‘open’ or ‘obstructed’. This is the first neural network model based on a DL algorithm to identify sinus CTs.It is not clear whether this image classification is predictive of clinical outcomes, but standardisation of image reporting and improved accuracy are valuable in their own right. 2022 Massey et al. (Massey et al., 2022) established that a CNN-based sinus CT assessment method was able to provide rapid and automated quantitative assessment of sinus turbidity and that this AI technique achieved good performance compared to current standard visual assessment systems. Segmentation of the sinuses is necessary as they are complex anatomical structures with highly variable shape and size, and their morphological and volumetric data can be used for diagnosis, surgical planning and simulation. However, when lesions such as pus, bone destruction, and Onodi’s airspace are present in the sinuses, their boundaries cannot be clearly visualised and segmentation is difficult. Therefore, in 2021 Qi et al. (Qi et al., 2021) proposed a CNN-based adaptive region localisation level set method that can be used for segmentation of diseased maxillary sinus. Compared with the methods of fast level set (FLS) and conditional random field - fully convolutional network (CRF-FCN), their Dice similarity coefficients (DSC) on average 0.25 and 0.12, respectively, obtaining significant improvements. In 2022, Choi et al. (Choi et al., 2022) trained a U-Net model to segment the maxillary sinus. The segmentation results were refined using post-processing techniques to isolate and eliminate disconnected false positives. The DSC value of the trained model was 0.90 ± 0.19 before post-processing and 0.90 ± 0.19 after post-processing. In the same year Morgan et al. (Morgan et al., 2022) trained two U-Net models to segment the maxillary sinus. The first model suggested the use of a crop frame in the original image of the maxillary sinus, which was used to train the second part of the model to produce high resolution segmentation results. The final segmentation results showed a DSC score of 0.98 for the first model and 0.99 for the second model. Both methods showed adequate performance for clinical applications. However, the aim of these two studies was limited to binary segmentation of the maxillary sinus. In 2024 Whangbo et al. (Whangbo et al., 2024) introduced a multi-class CNN segmentation model by comparing four 3D U-Net variants (normal, residual, dense and residual-dense). Data were normalised and trained on 40 patients (20 normal, 20 abnormal) using 5-fold cross-validation. In the normal test set, the model performance was in the range of 0.843–0.785 with a mean F1 score of 0.805. In the abnormal test set, the model performance was in the range of 0.793–0.740 with a mean F1 score of 0.755. True positivity was higher for the pterygoid and maxillary sinuses in both groups. The enhanced segmentation of abnormal sinuses by this algorithm suggests potential clinical applications.

In medical imaging diagnosis, ResNet supports ultra-deep networks with high classification accuracy, which is more suitable for high-precision classification tasks (such as disease classification), but it needs to set up additional decoders to perform segmentation tasks (He et al., 2016; Voinea et al., 2024). U-Net has high segmentation accuracy and is irreplaceable in segmentation tasks, but it is not suitable for classification tasks (Siddique et al., 2021). However, traditional CNNs are gradually replaced by more complex models due to their difficulty in capturing complex features and poor segmentation of medical images (Nirthika et al., 2022). In practical applications, multi-task collaboration is often achieved through model fusion (e.g., ResNet + U-Net) or improved structures (e.g., 3D U-Net) to promote the development of precision medicine (Ge et al., 2022; Alsaleh et al., 2024).

#### 3.1.2 Application of artificial intelligence in the pathological analysis of chronic sinusitis

CRS is a prevalent chronic inflammatory disease of the upper respiratory tract that affects individuals of all ages. The 2012 European Position Paper on Rhinosinusitis and Nasal Polyposis (EPOS) guidelines divided CRS into two main phenotypes, CRS with nasal polyps (CRSwNP) and CRS without nasal polyps (CRSsNP) (Fokkens et al., 2012), but the 2020 New EPOS guidelines divide primary CRS into type 2 and non-type 2 (Fokkens et al., 2020), namely, eosinophilic CRS (eCRS) and non-eosinophilic CRS (non-eCRS). The classification is based on the main inflammatory cell types observed in histopathological analyses.

Currently, the traditional approach to detecting endotypes relies heavily on pathological biopsies, which are often considered the baseline method. Manual identification and labelling of microvessels is labour-intensive and can be error-prone. Therefore, automated and accurate detection and quantification methods are essential (Table 2). In 2020 Wu et al. (Wu et al., 2020) used DL algorithms to build an artificial intelligence evaluation platform (AI CRS Evaluation Platform [AICEP]), which was used to diagnose nasal polyp pathology types by whole slide imaging (WSI) with high sensitivity and AUC values. However, AICEP 1.0 could only differentiate between eCRSwNP and non-eCRSwNP and was unable to obtain the proportion of each inflammatory cell on the WSI. In 2021, Wu et al. (Wu et al., 2021) established another AI Chronic Sinusitis Evaluation Platform 2.0 (AICEP 2.0), which extends the previous AICEP 1.0 b y further analysing the cellular phenotypes of nasal polyps, and allows the distribution of the concentration of the four types of inflammatory cells in the WSI to be predicted by heat maps with different prognoses. In addition, this method demonstrated for the first time that the percentage of peripheral blood eosinophils positively correlates with the percentage of eosinophils in polyp tissue on WSI and predicts whether a patient is an eCRSwNP. 2022 Liu et al. (Liu et al., 2022) used a fully convolutional neural network (FCN) model to detect and quantify microvessels in the human nasal mucosa, and the quantification of microvessels in type 2 and non-type 2 CRS showed considerable differences, with higher expression in type 2 CRS.

**TABLE 2:** The role of artificial intelligence in the pathological analysis of chronic sinusitis.

Researcher	Year	System	Function	AI	Ref
Wu	2020	Inception V3	To diagnose eCRSwNP rapidly and accurately via WSI	DL	Wu et al. (2020)
Wu	2021	EfficientNet B5	Cellular phenotyping diagnosis of nasal polyps by WSI based on the proportions of inflammatory cells	DL	Wu et al. (2021)
Liu	2022	FCN	To explore the tissue quantification of microvessels and their potential association with inflammation in CRS	CNN	Liu et al. (2022)

CRS, chronic sinusitis; eCRSwNP, eosinophilic chronic sinusitis with nasal polyps; WSI, whole slide imaging; DL, deep learning; FCN, fully convolutional neural network; CNN, convolutional neural network.

### 3.2 Artificial intelligence in the treatment of chronic sinusitis

The current treatment of CRS mainly includes medication and surgery, and immunotherapy also has a very good prospect of application. In the field of clinical practice, it has been observed that different endotypes exhibit different diagnostic and therapeutic approaches, with surgical treatment playing a crucial role. Functional endoscopic sinus surgery (FESS) prioritises the preservation of mucosal tissue and is considered more suitable for non-eCRS cases, with eCRS having a recurrence rate of up to 98.5% (Cardell et al., 2020). However, surgical procedures for eCRS patients with significant inflammatory load require more mucosal management to reduce the inflammatory burden, such as extended endoscopic sinus surgery and Draf III surgery, which has a wider surgical area than non-eCRS surgery (McHugh et al., 2018; Bachert et al., 2020). It highlights the need for accurate diagnosis of CRS endotypes to facilitate the development of personalised and targeted therapies. However, clinical acquisition of CRS pathological endotypes usually occurs in the postoperative period and it is not possible to identify inflammation and determine endotypes during the surgical and perioperative period. Therefore, accurate identification of CRS endotypes prior to surgical intervention can assist clinicians in developing appropriate surgical strategies to reduce postoperative recurrence rates, which is essential for individualised treatment of chronic rhinosinusitis (Table 3).

**TABLE 3:** Application of artificial intelligence in predicting endotypes of chronic sinusitis.

Researcher	Year	Model	Input	Function	AI	Ref
Thorwarth	2021	LR,ANN	PEC,uLTE4, polyp status	Diagnosis of eCRS	ML	Thorwarth et al. (2021)
Xiong	2024	LR with lasso regression,RF, GBDT,DNN	peripheral blood eosinophil ratio, absolute peripheral blood eosinophil, E/M	Prediction of eCRS	ML	Xiong et al. (2024)
Hua	2023	U-net,Deeplabv3, efficientnet-b0, ResNet-50, Inception-ResNet-v2,Xception	Preprocessed axial spiral CT images	Differentiation of eCRS and non-eCRS on preoperative CT	DL	Hua et al. (2023)
Du	2024	ResNet-18	PNS CT	Prediction of CRSwNP endotypes	DL	Du et al. (2024)
Zou	2024	ResNet mini	three-view pictures of sinus CT scans	CRSwNP endotype identification	DL	Zou et al. (2024)

LR, logistic regression; ANN, artificial neural network; PEC, peripheral eosinophil count; uLTE4, urinary leukotriene E4; ML, meachine learning; eCRS, eosinophilic chronic sinusitis; RF, random forest; GBDT, gradient-boosted decision tree; DNN, deep neural network; DL, deep learning; E/M, the ethmoidal/maxillary sinus density ratio; non-eCRS, non-eosinophilic chronic sinusitis; PNS, paranasal sinus; CRSwNP, chronic rhinosinusitis with nasal polyps.

In 2021, Thorwarth et al. (Thorwarth et al., 2021) developed a logistic regression (LR) and artificial neural network (ANN) machine learning model to predict eCRS by inputting variables such as peripheral eosinophil counts, urinary leukotriene E4 (uLTE4) levels, and polyp status, and the AUCs for the logistic regression model were 0.882 and 0.945. The AUCs for the ANN model were 0.918 and 0.956, respectively. The logistic regression and ANN models were not statistically different when compared. It is possible to predict eCRS with high sensitivity and specificity in this patient population.

In 2024, Xiong (Xiong et al., 2024) and others developed a prediction model for eCRS based on patient clinical parameters using algorithms such as logistic regression with lasso regularisation, random forest (RF), gradient-enhanced decision tree (GBDT), and deep neural network (DNN), which identified the peripheral blood eosinophil ratio, absolute peripheral blood eosinophil value, and the sieve bone/maxillary sinus density ratio (E/M) on CT as key predictors of eCRS.

The predictive models provide a valuable tool for identifying eCRS without resorting to histological biopsy, thus enhancing clinical decision-making. However, the variables entered into these models still require invasive manoeuvres to obtain blood specimens, so reliable non-invasive methods to identify endotypes of CRSwNP are needed, and imaging histology is also of good value in this regard.

2023 Hua et al. (Hua et al., 2023) who constructed a prediction model for CRS endophenotypes based on sinus CT images using U-net and other neural networks, had good accuracy in predicting image endophenotypes and patient endophenotypes with AUC values of 0.762 and 0.853, respectively. In 2024 Du et al. (Du et al., 2024)who first used ResNet-18 to construct a deep learning model to differentiate and predict the intrinsic type of CRSwNP, which predicted all patients with CRSwNP with an AUC of 0.962,and had good predictive performance in patients with eCRSwNP and non-eCRSwNP, with AUCs of 0.960 and 0.964. In the same year Zou et al. (Zou et al., 2024) proposed a multi-view DL fusion classification model for the diagnosis of CRSwNP endotypes using sinus CT scans. The multi-view perspective model improves performance by integrating sinus CT axial, coronal and sagittal image data to effectively utilise the information. The model achieved a maximum accuracy of 96.54% on the test set and an AUC value of 0.991.

Also due to the complexity and individual variation of the anatomical region of the paranasal sinuses, there are many important anatomical structures in the paranasal sinuses and surrounding tissues, and there is much variation in these structures, and the identification of clinically important structures in sinus surgery is essential to reduce surgical complications. In 2020, Huang et al. (Huang et al., 2020) used a CNN algorithm to differentiate the location of the anterior sieve artery in sinus computed tomography with an overall accuracy of 82.7% and an AUC value of 0.86. In the same year Parmar et al. (Parmar et al., 2020) trained a CNN algorithm appeared to be successful in identifying pneumatisation of the middle turbinate with high accuracy. The diagnostic accuracy was 81% (95% confidence interval: 73.0%–89.0%) with an AUC of 0.93. These two models provide a good application idea for clinical AI to identify important anatomical variants in rhinology to guide sinus surgery to reduce surgical complications.

The integration of AI with navigation systems has revolutionised the way surgeons conduct surgery. Traditional navigation relies on static pre-operative images that may not accurately represent intraoperative changes, which can lead to discrepancies between planned and actual surgical paths. Physicians often need to manually update conventional navigation systems when a patient’s positioning or anatomy changes. This can be time-consuming and may introduce errors. Traditional navigation provides mainly spatial guidance and lacks real-time dynamic information about key structures and their relationships. AI-based navigation integrates advanced machine learning, deep learning algorithms, and real-time data processing (Neves et al., 2021). The AI algorithms analyse intraoperative images and sensor data to provide dynamic guidance throughout the procedure. AI-based navigation has the advantage of real-time adaptation. AI algorithms continuously analyse intraoperative images, provide real-time updates and adapt to changes in the surgical field. This dynamic adaptation improves accuracy and reduces the risk of error. AI algorithms can alert physicians to potential complications, such as excessive tissue manipulation or the proximity of instruments to sensitive areas, so that corrective action can be taken in a timely manner (Sekhar et al., 2020).

Type 2 inflammation is associated with comorbidities such as asthma, leading to increased disease severity and morbidity compared to non-type 2 inflammation, and therefore patients with type 2 inflammation require more surgical procedures and extensive medical interventions. To address CRS with uncontrolled type 2 inflammation, new biologics, such as monoclonal antibodies, are available. However, the lack of tests to assess molecular biomarkers hinders personalised medicine for patients with CRS. The prescribing criteria for biologically targeted therapies in patients with CRSwNP are largely dependent on clinical and histological/blood test results (Fokkens et al., 2020).2024 Federico Sireci et al. (Sireci et al., 2024)assessed the concordance between ChatGPT and the Rhinology Committee’s recommendations for the use of biologic therapies for the treatment of patients with CRSwNP. Observations highlighted the potential of ChatGPT in guiding the optimal choice of biologic therapy, with a percentage of concordance was 68% and a Kappa coefficient was 0.69 (CI95% [0.50; 0.75]). In particular, the concordance was 79.6% in the dupliyuzumab group, respectively.

### 3.3 Application of artificial intelligence in determining the prognosis of chronic sinusitis

Although CRS after endoscopic sinus surgery usually has a high initial success rate, its postoperative recurrence has always been a headache for rhinologists and patients (Hopkins et al., 2009). The success rate of ESS ranges from 76% to 98%, which is usually associated with ESS Common failure factors include inappropriate surgical technique, poor surgical area or visualisation, and inadequate postoperative care (Chang et al., 2014). According to a large prospective cohort study, approximately 20% of patients are dissatisfied with their surgical response and require revision during the 5-year follow-up period. In this study, 20.6% of patients with polyps had undergone revision surgery within the past 5 years, compared with 15.5% of patients with CRS alone (Hopkins et al., 2009).

In 2021, Wang et al. (Wang et al., 2021)scholarly study explored the combined effect of non-invasive clinical markers on the recurrence of CRSwNP, using a ML algorithm to assess the predictive value of a history of asthma and percentage of blood eosinophils, and the results showed that For patients with CRSwNP with asthma, the critical value of percentage of blood eosinophils was 3.7%. However, for CRSwNP patients without asthma, the critical value of blood eosinophil percentage was high at 6.9%. It was confirmed that the combination of history of asthma and blood eosinophil percentage predicted CRSwNP recurrence, whereas history of asthma lowered the threshold of blood eosinophil percentage to predict CRSwNP recurrence. In 2022 Yu et al. (Yu and Kim, 2022) constructed 3 ML prediction models, decision tree (DT), random forest (RF) and support vector machine (SVM), and the validation analysis showed that the RF algorithm had the highest F1 scores and AUC. The model demonstrated that increased neutrophilic inflammation in patients with refractory CRSwNP, and an increase in neutrophils in the subepithelial region was associated with the patients with CRSwNP with poor surgical outcomes. 2022 Nuutinen et al. (Nuutinen et al., 2022) identified individual-level risk factors associated with revision of the ESS in patients with CRS by building a predictive model using machine learning algorithms. Type 2 hyperresponsive disorders (CRSwNP, asthma and non-steroidal anti-inflammatory drugs (NSAIDs) aggravated by respiratory disease NERDs), a high frequency of clinical visits, short intervals between baseline clinic visits and ESS, and immune deficiency or suspected immune deficiency increased the likelihood of individual-level revision of the ESS. and immunodeficiency or suspected immunodeficiency increase the likelihood of ESS revision at the individual level.

## 4 Deficiencies and recommendations for artificial intelligence in current research

Previous studies have shown that CRS is a chronic disease with a high degree of individual variation. For patients, the severity and staging of the disease vary, as do the corresponding treatment options and prognosis. For physicians, imbalances in healthcare resources and differences in understanding of disease may also affect the choice of treatment options, leading to variations in patient outcomes. Standardised treatment protocols for CRS are usually based on clinical practice guidelines and clinical best practice to provide a uniform and standardised approach to the treatment of patients with CRS. Therefore, optimising standardised CRS care is a very challenging and significant area of research.

In recent years, with the development of AI, its research in the diagnosis, treatment and prognosis of CRS has been increasing with good results. AI is able to analyse and process massive amounts of data and train algorithmic models with this processed information to accurately identify the information detected and arrive at a clinically appropriate diagnosis. This can, to a certain extent, solve the problem of different diagnostic conclusions due to differences in the level of different diagnostic doctors, and can greatly improve the efficiency of diagnostic doctors. At the same time, AI can assist rhinologists in making clinical decisions and developing personalised treatment plans. In a sense, AI extends the human organ, deepens human knowledge and understanding of chronic sinusitis, and provides new ideas for future treatment modalities of CRS.

Although AI has shown great potential in healthcare, to use it to optimize the standardization of CRS diagnosis and treatment, it is important to validate the safety and efficacy of AI models in the clinic from a real-world testing and regulatory approval perspective. Most current studies of AI applied to CRS are single-center retrospective studies. The data used in these studies lacked standardization and were less reproducible. Moreover, there are differences in different AI models, resulting in similar data of varying quality. At the same time, it is difficult to share data because of the relative independence of each healthcare organization. The variable quality of data can affect the accuracy of model interpretation results, leading to a decrease in diagnostic accuracy (Wang et al., 2024). Therefore, it is necessary to establish a systematic and comprehensive standardized database to train AI models. The database needs to cover different regions, populations and devices collecting data to avoid insufficient model generalization capability due to data bias. Screening and constructing effective datasets, building a mature data sharing platform, and establishing a perfect data standard system to ensure data security and maximum sharing. Meanwhile, it is necessary to supplement the insufficiency of retrospective studies, conduct prospective randomized controlled trials (RCTs), verify the generalizability of the model through cross-institutional collaboration, further improve the performance of the AI model, and adhere to long-term follow-up to verify the actual clinical benefits.

The clinical acceptance of AI is also a matter of great concern. Many AI models (such as ML) are “black box” models, resulting in the unexplainability of their internal operations, making it difficult for doctors and patients to understand their decision-making process, difficult to ensure the credibility and safety of results, and reducing the trust and acceptance of AI system models by doctors and patients. In view of this, research in recent years has focused on the development of new explainable artificial intelligence (XAI) technologies (van der Velden et al., 2022; Zhang et al., 2022; Liu et al., 2024; Sarkar et al., 2024), such as lime, SHAP (Rahimi et al., 2023; Yagin et al., 2023), etc.,. XAI can capture the results and outputs of ML/DL algorithms, provide model decision-making and interpretation to overcome the limitations of the black-box nature of artificial intelligence, and show great promise in diagnostics and drug discovery and development prediction (Zhang et al., 2022; Ali et al., 2023; Kırboğa et al., 2023). However, at present, XAI technology is still in the stage of exploration and development, and has not been effectively and comprehensively standardized and evaluated.

We also have to continually evaluate AI systems to ensure that they work as intended, remain accurate over time, and are reliable for medical purposes. Medical knowledge is rapidly updated. Therefore continuous monitoring and evaluation of AI algorithms is essential to maintain their effectiveness, accuracy, and reliability (Kalpathy-Cramer et al., 2021). AI models need to learn continuously to adapt to real-world dynamics (e.g., the evolution of disease profiles or the introduction of new detection technologies) to avoid “model degradation”. This requires that AI algorithms are also up-to-date, and therefore algorithmic models should be regularly evaluated to check the accuracy of the generated content and to ensure that the information remains up-to-date and consistent with current medical knowledge (Knopp et al., 2023). In addition, monitoring the performance of the algorithm can also help to identify any potential biases or unintended consequences that may arise during its use (Larson et al., 2021).

Similarly, the ethical and patient safety issues raised by AI cannot be ignored. The establishment of specialized ethics committees is essential to proactively address any ethical issues that may arise when applying AI in healthcare (McKay et al., 2023). Ethics committees play a critical role in emphasizing the importance of regulatory compliance and reviewing potential ethical challenges, including privacy issues, fairness and transparency (Abràmoff et al., 2022). The use of data needs to comply with relevant information security protection regulations, protect patient privacy, ensure compliant use of data, and an adverse reaction reporting system needs to be set up to dynamically regulate AI models once they are on the market. In addition to the regulatory aspects, the role of ethics committees involves legal issues that extend into the realm of liability, as well as the impact of AI decision-making, especially when problems arise (Vidalis, 2021). Currently, AI is used as an auxiliary tool, and the diagnostic results need to be reviewed by a physician, for example, the artificial intelligence cervical cancer screening (AICCS) system requires the screener to compare the results with the AI results, and in case of conflict, the pathologist will adjudicate to ensure that the responsibility for the final decision is clear. If the AI makes autonomous decisions in the future, legislation is needed to clarify the division of responsibility (Wang et al., 2024). At the same time, organizations must be vigilant, informed and compliant with regulations and standards when integrating AI into healthcare (Schulz et al., 2019). These organizations should keep abreast of healthcare data protection laws and ensure that AI systems comply with these regulations (Stanfill and Marc, 2019). Regular updates and compliance checks should be conducted to adapt to changes in the law in order to maintain legal and ethical integrity in the use of AI in healthcare (Wolf, 2020).

To verify the clinical applicability of AI models, it is necessary to build a “data-test-regulation” closed loop, ensure model robustness through real-world testing, balance innovation and risk through dynamic regulation of models, and ultimately realize the transition from an assistive tool to a credible decision-making role. In the future, the focus needs to be on issues such as defining responsibility and algorithmic transparency to drive AI models from research to real-world clinical applications.

**FIGURE 2:**
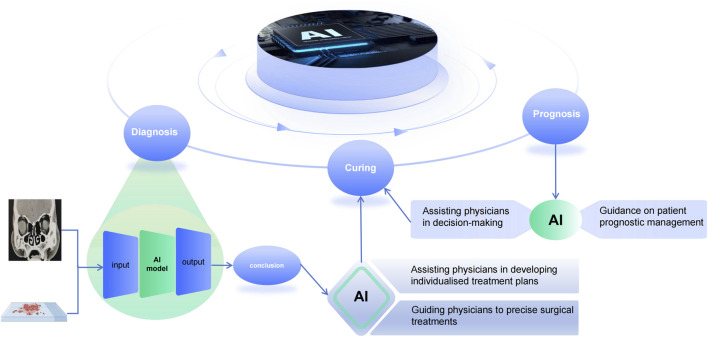
Artificial intelligence in the diagnosis, treatment and prediction of prognosis of chronic sinusitis. Inputting image pictures and pathology results to the AI system yields an accurate diagnosis, guiding doctors to formulate individualized treatment plans for patients, and at the same time, the prognosis predicted by the AI-assisted system can assist doctors in making decisions about the management of patients’ conditions.

The application of AI technology to the management of CRS is of great significance, but this does not mean that AI will replace clinicians. On the contrary, it is a new model that integrates artificial intelligence and human beings. This new model can effectively promote the intelligence, standardisation and standardisation of chronic sinusitis diagnosis and treatment. Although the application of existing AI technology in healthcare is still immature, and most of the current research is in the area of diagnosis. However, we need to work together with relevant technicians to create a better blueprint for the future ‘AI + Healthcare’ model (Figure 2).

## 5 Conclusion

With the continuous development of AI,it is gradually playing an increasingly important role in healthcare. AI can significantly improve the accuracy of disease diagnosis, the level of personalized treatment, and the efficiency of medical resource utilization. The diagnosis and treatment mode of disease is also changing from the traditional diagnosis and treatment mode to the mode of ‘AI + medicine’. There are more and more studies on the application of AI to CRS, but there are also many challenges. Issues such as data quality, privacy, ethics, regulations, technical limitations, and clinical acceptance still need to be addressed. Nevertheless, AI has already proved its excellence in the medical field through continuous development. It is expected that with the advancement of technology and the improvement of regulations in the future, artificial intelligence can bring more surprises to doctors and patients.
